# Comparative Analysis of Two Dietary Saturated Fat Types on Metabolite Profiles Crossing the Blood–Brain Barrier of Poultry Chicks

**DOI:** 10.3390/metabo16040283

**Published:** 2026-04-20

**Authors:** Oluteru E. Orimaye, Paul C. Omaliko, Nathanael I. Lichti, Bruce R. Cooper, Yewande O. Fasina

**Affiliations:** 1 Department of Animal Sciences, North Carolina Agricultural and Technical State University, Greensboro, NC 27411, USA; oeorimaye@aggies.ncat.edu (O.E.O.); pcomaliko@aggies.ncat.edu (P.C.O.); 2 Bindley Bioscience Center, Purdue University, West Lafayette, IN 47907, USA; nlichti@purdue.edu (N.I.L.); brcooper@purdue.edu (B.R.C.)

**Keywords:** coconut oil, hypothalamus, dorsal raphe nucleus, metabolomics, metabolites

## Abstract

**Background:** The dorsal raphe nucleus (DRN) produces and distributes serotonin, while the hypothalamus (HYP) uses serotonergic signals to regulate physiological processes in chickens. Coconut oil (COCO), rich in medium-chain fatty acids, is rapidly absorbed without re-esterification. **Methods:** Day-old broilers (Ross 708 male, n = 160) were distributed into two dietary treatments with five replicates of 16 birds each. The birds were fed a corn–soybean meal (SBM) basal diet supplemented with 3% of poultry fat (CON) or coconut oil (COCO). The body-weight gain (BWG), feed intake (FI), and feed conversion ratio (FCR) were recorded over a 3-week period, and the data were subjected to a *t*-test. Untargeted metabolomic analysis by high-performance liquid chromatography (HPLC-MS) was used to evaluate the influence of the type of dietary fat on metabolite profiles in the DRN, HYP, and plasma of broiler chickens. Principal component analysis (PCA) was used to identify unique metabolites, and ANOVA was used to identify the metabolites that were significantly abundant (*p* < 0.05). The metabolites were then annotated using the KEGG and HMDB databases. **Results:** Birds in the COCO treatment gained more weight on average (0.8446 kg/bird) than birds in the CON group (0.8132 kg/bird; *p* = 0.0496). Five metabolites associated with multiple significant cellular processes, such as brain function, energy metabolism, and neurotransmission, showed similar differential expression patterns, while two metabolic pathways (butanoate metabolism and alanine, aspartate and glutamate metabolism) were identified. **Conclusions:** The dietary inclusion of COCO improves BWG in poultry and enhances their overall well-being by modulating metabolite profiles, supporting neurotransmission, and enriching the metabolic pathways essential for growth and brain function.

## 1. Introduction

The blood-brain barrier (BBB) plays a critical role in maintaining brain homeostasis by regulating the passage of nutrients, toxins, and immune cells into the brain, while dietary fats are essential nutrients crucial for various physiological functions, including storing energy, supplying essential fatty acids, absorbing fat-soluble vitamins, protecting organs, and acting as precursors to steroid hormones. Saturated fatty acids comprise three sub-groups: short-chain (C2–C6), medium-chain (C6–C12), and long-chain (C14–C24). Long-chain fatty acids, found in animal fats such as tallow and lard, exhibit significant variability in their composition.

All three sub-groups are thus grouped in interpreting diet and disease relationships and making diet recommendations. There is a growing demand for feeding broiler chicks plant-based fats as an alternative to animal fats, but the cost is a major determinant of their utilization under a certain commercial scenario. According to Crespo and Esteve [1], the broiler-fed diets supplemented with olive oil, sunflower oil, and linseed oil exhibited lower levels of saturated fatty acid (FA) in their breast muscle, thigh muscle, and abdominal fat pad than chickens fed meals containing tallow. Dietary fatty acids are directly incorporated into adipose tissues, which results in the observed alterations in fatty acid composition [1].

Across the world, coconuts have long been used as food and medicine, and over four thousand years ago, their application in Ayurvedic medicine was recorded [2]. Coconut oil (COCO) is made up of about 92% saturated fats and 9% unsaturated fats. However, the saturated fats in plants differ from the saturated fats in animal tissues. Medium-chain fatty acids (MCFAs) are believed to disrupt signal transduction, as well as the assembly and maturation of viruses [3], by breaking down their lipid membrane and helping to eliminate various lipid-coated bacteria [4].

It is important to study the effect of COCO on compounds that might cross the BBB in broiler chickens, and optimizing the BBB integrity is essential for neurological health, cognitive function, and overall performance of broiler chicks.

Recently, interest has been directed towards the dietary incorporation of COCO as a result of its high concentration of MCFAs, which may positively influence BBB permeability. The fats in COCO are 65% medium-chain and predominantly (~48%) lauric acid (C12), which possess neuroprotective properties [5]. These fatty acids can serve as an alternative energy source, providing ketone bodies that support brain metabolism [6]. Additionally, COCO has been shown to modulate lipid profiles, reducing oxidative stress and inflammation, which are two major factors that compromise BBB integrity [7]. Omaliko et al. [8] suggested that dietary supplementation with COCO enhances the expression of tight junction proteins such as occludin and claudin-5, which are vital for maintaining the BBB structure and function [8]. Furthermore, the antimicrobial properties of COCO may be used to control systemic infections that could otherwise weaken the BBB. There is not enough research to support the potential benefits of COCO in maintaining BBB integrity in poultry production. Understanding the role of COCO in BBB function could open new avenues for enhancing poultry health and performance through dietary interventions, as nutritional strategies are known to modulate the gut–brain axis and barrier functions [9].

In this study, we are studying the effect of COCO on compounds that might cross the BBB to the serotonergic-rich sections of the brain, specifically the DRN and the HYP, as well as the plasma and broiler growth performance.

## 2. Materials and Methods

The animal care and use procedures were approved by the Institutional Animal Care and Use Committee (IACUC 20-004.0) of North Carolina Agricultural and Technical State University.

### 2.1. Experimental Design, Diet, and Bird Management

Day-old broilers (Ross 708 male, n = 160) were sourced commercially and housed at the Poultry Research Unit of the North Carolina Agricultural and Technical State University (Greensboro, NC, USA) during a 3-week experiment. The birds were distributed into two dietary treatments with five replicates of 16 birds each. The rearing and management of the chicks followed Aviagen’s standard protocols for the Ross 708 strain. The experimental treatments (Table 1) included a basal diet of corn–soybean meal (SBM) with a 3% inclusion of dietary fats: poultry fat (CON) and coconut oil (COCO). The experimental diet was calculated to be equicaloric and was manufactured at the North Carolina State University Feed Education Unit (Raleigh, NC, USA). The type of oil used was procured commercially from Edwards International, Inc. (Braintree, MA, USA). Throughout the experiment, the birds had unrestricted access to the experimental diets, which were provided as pellet crumbles to promote optimal feed intake and growth performance. From d 1 to d 21, the chicks were housed in battery cages (Alternative Design Manufacturing and amp Supply Inc., Siloam Springs, AR, USA). Each battery cage was equipped with a nipple drinker to provide water and a feeder tray that was adjusted according to the growth of the chicks. Room temperature was maintained at 92 °F from day 1 to 7 and reduced to 87 °F from day 8 to 21. A photoperiod of (23L:1D) was applied from placement to day 7, followed by (20L:4D) from day 8 to 21.

### 2.2. Growth Performance Indices

The performance indices for the body weight (BW), body-weight gain (BWG), and feed intake (FI) of the chicks were recorded weekly for the assessment of growth performance. From the resulting data, the feed conversion ratio (FCR) was calculated.

### 2.3. Sample Preparation for LC-MS

Here, 100 mg of each sample tissue (DRN and HYP) was weighed, separately thawed on ice, and homogenized using a Precellys 24 tissue homogenizer (Bertin Tech., Montigny-le-Bretonneux, France) at 2500 rpm for 15 s. After homogenization, 1000 µL of 80% methanol was added, and the samples were re-homogenized to ensure a thorough extraction of metabolites. The homogenates were then transferred to prelabeled microcentrifuge tubes and centrifuged at 10,000 rpm for 8 min using a Sorvall Legend Micro 21 centrifuge (Thermo Scientific, Osterode am Harz, Germany). The resulting supernatant was carefully collected into new prelabeled microcentrifuge tubes, while the pellets were discarded. To remove the solvent, the supernatants were evaporated overnight in a SAVANT SPD2010 SpeedVac concentrator (Thermo Fisher Scientific, Asheville, NC, USA). The dried extracts were reconstituted by adding 75 µL of a solution containing 95% water, 5% acetonitrile, and 0.1% formic acid. The reconstituted samples were sonicated for 5 min to ensure complete dissolution and then centrifuged at 13,000 rpm for 8 min. The clear supernatants were transferred to Waters HPLC vials for analysis, with pooled quality control samples prepared by combining aliquots from all samples to monitor system performance throughout the analytical run.

### 2.4. Untargeted Metabolomic Analysis Using HPLC-MS

The LC-MS analysis was performed using an Agilent 1290 Infinity II liquid chromatography system and an Agilent 6546 series QQQ mass spectrometer (Agilent Tech., Yishun, Singapore). The Atlantis T3 column was used for LC separation, with buffers of water + 0.1% formic acid and acetonitrile + 0.1% formic acid. Multiple reaction monitoring was used for MS analysis, with data acquired in positive electrospray ionization (ESI) mode. The jet stream ESI interface had various conditions, including gas temperature, flow rate, nebulizer pressure, sheath gas temperature, capillary voltage, and nozzle voltage. The injection volume for each sample was 4 μL. A high-resolution Agilent mass spectrometer, TripleTOF 6546 LC/Q-TOF (Agilent Tech., Yishun, Singapore), was used to detect metabolites eluted from the column. The Q-TOF was operated in both positive and negative ion modes, with the curtain gas set at 30 PSI, ion source gas1 at 60 PSI, ion source gas2 at 60 PSI, and the interface heater temperature at 650 °C. The mass spectrometry data were acquired in IDA mode, with the TOF mass range from 60 to 1200 Da. Survey scans were acquired in 150 ms, and up to 12 product ion scans were collected if a threshold of 100 counts per second was exceeded and a 1+ charge-state was detected. The total cycle time was fixed to 0.56 s, and four times bins were summed for each scan at a pulse frequency value of 11 kHz. Dynamic exclusion was set at 4 s. Mass accuracy was calibrated every 20 samples, and a quality control sample was acquired after every 10 samples to evaluate the stability of the LC-MS system.

### 2.5. Processing of Metabolite Data

MS-DIAL version 4.9 was used to perform pretreatments on the acquired MS data, including peak detection, peak grouping, alignment, gap filling, retention time correction, second peak grouping, and annotation of isotopes and adducts. The intensity of the peak data was further pre-processed by MS-DIAL, and features detected in less than 60% of QC samples were removed. NS-KNN imputation of Lee and ref. [10] was used to impute missing values. Principal component analysis (PCA) was performed for outlier detection and batch effects evaluation. The data in the CSV files were exported for further analysis. Online open-source KEGG and HMDB [11] databases were used to annotate metabolites by matching the exact molecular mass data of samples with those from the database.

### 2.6. Statistical Analysis

Intensities were log-10 transformed prior to analysis. Multivariate analysis involving PCA was used to reduce data dimensionality and visualize sample clustering and separations based on the metabolite profiles. Univariate linear models (two-way ANOVA) using conditional contrasts of treatments within tissues were used to identify metabolites that differed significantly between treatments. This approach assumes that substantial differences in expression will exist among tissues and therefore focuses specifically on the differences between COCO and the CON within each tissue, rather than differences across tissues. Statistical analyses were performed in R, version 4.4.1 [12], with contrasts calculated using the emmeans package 2.0.3 [13]. The online metabolite pathway enrichment program MetaboAnalyst 5.0 [14,15] was used to conduct metabolite pathway analysis.

## 3. Results

### 3.1. Treatment Effects on Broiler Performance

Results generated for this study using a *t*-test showed that the BWG was significantly higher in the COCO group compared to the CON (Table 2). BW and FI trended higher with COCO, but were not significant, while FCR did not differ, indicating a similar feed efficiency between diets.

### 3.2. Metabolomic Profiling and Tissue-Specific Signatures

**Figure 1:**
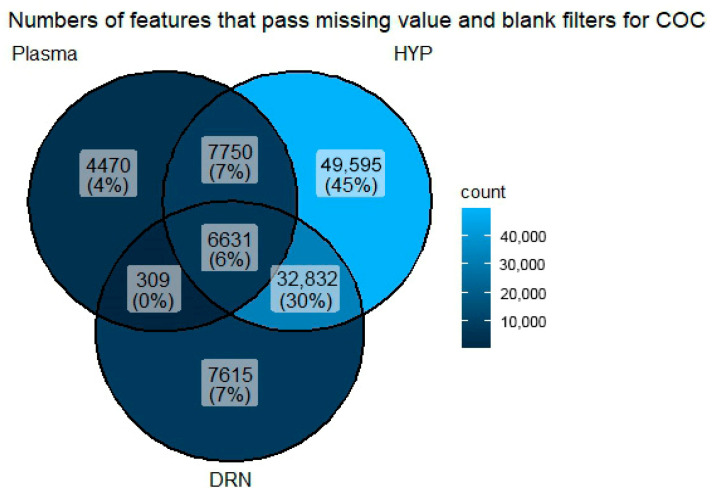
Venn diagrams showing overlaps of differentially expressed values across the plasma, DRN, and HYP.

The Venn diagram in Figure 1 illustrates the distribution of metabolomic features that passed the missing value and blank filters across tissue types: the plasma, the HYP, and the DRN. A total of 110,182 features were detected, with distinct and overlapping patterns across the sample types. The HYP exhibited the highest number of unique features, with 49,595 features (45%) exclusively present in this tissue. A substantial number of features (32,832; 30%) were shared between the HYP and DRN, implying a notable biochemical overlap between these two brain regions, possibly due to shared neurochemical functions or regulatory pathways. In contrast, 7750 features (7%) were shared between the plasma and the HYP, while 7615 features (7%) were unique to the DRN, highlighting the DRN’s specific metabolomic profile. Only 6631 features (6%) were commonly detected across all three tissue types, indicating that a relatively small core set of metabolites is conserved systemically and centrally. This common subset may represent baseline metabolic components that are essential across tissues. Other intersections showed minimal overlap: 4470 features (4%) were unique to the plasma, and only 309 features (0%) were shared between the plasma and the DRN exclusively, emphasizing the metabolic divergence between the peripheral (plasma) and the central nervous system (DRN and HYP). This result demonstrates distinct metabolic signatures among the three tissues, with HYP contributing the greatest number of unique and shared features. The minimal overlap between the plasma and the brain regions also highlights the importance of direct tissue analysis for accurate neurochemical profiling.

### 3.3. Principal Component Analysis (PCA)

**Figure 2:**
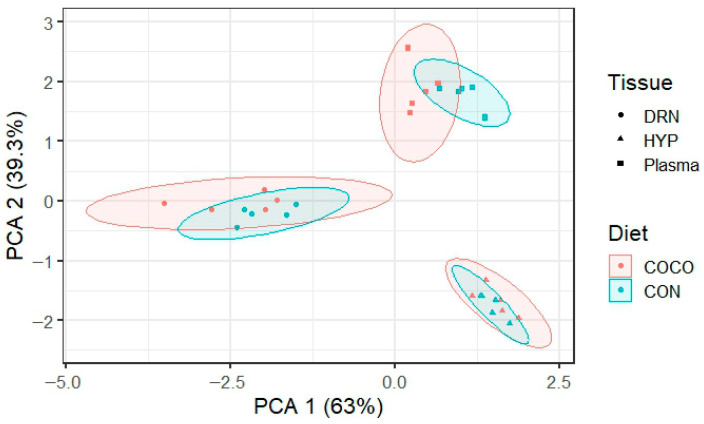
The PCA showing the distribution of metabolites.

Principal component analysis (PCA) was used to assess global metabolic differences across the plasma, HYP, and DRN under two dietary conditions: control (CON) and treatment (COCO) (Figure 2). The first two principal components, PC1 (63%) and PC2 (39.3%), summarize sample variability and group clustering. The PCA shows clear separation by tissue type, with plasma samples showing the most distinct separation between the CON and COCO, indicating a stronger systemic metabolic response to the diet. The overlap in HYP and DRN suggests more subtle, region-specific changes. These results highlight both the broad systemic impact of dietary intervention and the tissue-specific metabolic adaptations.

### 3.4. Tissue Metabolic Profiling and Non-Targeted Metabolite Dataset

In the present study, untargeted metabolomic analysis by high-performance liquid chromatography (HPLC-MS) was used to evaluate the influence of dietary fat types on metabolite profiles in the DRN, HYP, and plasma. Metabolites identified as being differentially expressed using ANOVA (*p* < 0.05) and were then annotated using the KEGG and HMDB databases. A total number of 5193 features were detected, 519 of which were annotated, and 49 of those were differentially expressed. Succinate semialdehyde, a critical metabolic intermediate that influences brain function, energy metabolism, and neurotransmitter regulation, was downregulated in the DRN. Pleiocarpamine, which possesses anticholinergic properties, was upregulated in the DRN, while both 2-Phenylethyl 3-O-β-D-glucopyranoside and methyl 4-oxy-2-hydroxy-3,6-dimethylbenzoate were downregulated in the HYP (Table 3). These identified compounds covered a wide range of biochemicals that include organic acids, amino acids, lipids, carbohydrates and benzenoids. These metabolites are involved in multiple significant pathways aiding key cellular processes.

### 3.5. Metabolite Set Enrichment Analysis (MSEA) and Pathway Modulation

**Figure 3:**
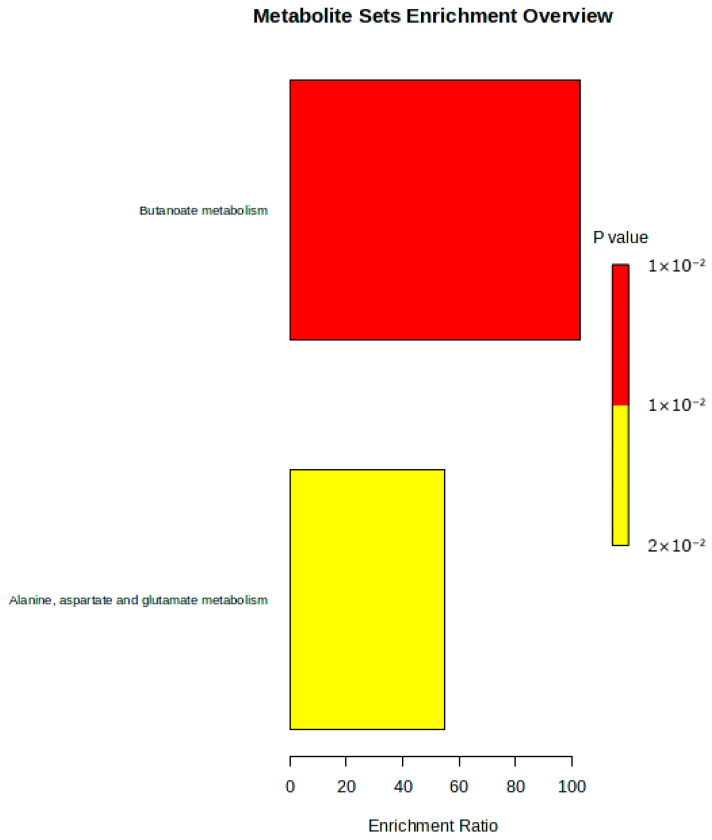
The metabolite set enrichment overview.

Using the KEGG database, only two pathways (butanoate metabolism and alanine, aspartate and glutamate metabolism) were generated, indicating a potential metabolic shift due to dietary or experimental interventions. The butanoate metabolism was the most significantly enriched pathway, represented by the largest red square, which indicates a high statistical significance (Figure 3). The alanine, aspartate, and glutamate metabolism pathway had an enrichment ratio of approximately 40 and a slightly higher *p*-value (~0.02), represented by a smaller yellow bar. This pathway enhances the potential impact of dietary intervention on amino acid turnover and neurotransmitter cycling. The extent to which these pathways are represented among the significantly altered metabolites suggests that it is the fraction of differentially expressed metabolites that are associated with a given pathway (Table 4).

## 4. Discussion

This study investigated the impact of COCO supplementation on broiler performance and metabolomic profiles in different tissues. The results revealed that birds fed the COCO diet exhibited a higher BWG when compared with the CON group. The enhanced BWG in the COCO group might be a result of the unique nutritional profile of COCO, which is rich in MCFAs such as lauric, capric, and caprylic acids [5,6]. These fatty acids are rapidly absorbed and metabolized to produce ketone bodies, thereby providing an immediate energy source that may promote growth. Similar observations have been reported by [22] where dietary fats with high MCFAs enhanced energy utilization and growth performance in poultry [22,23]. Despite these improvements in growth, there was no significant (*p* < 0.05) difference in FCR between the CON and the COCO. This suggests that while the birds gained weight more rapidly with COCO, the feed consumed was used with similar efficiency as the CON diet. It is possible that the improvement in BWG might be primarily due to enhanced nutrient absorption or metabolic shifts rather than improved feed conversion. Olsen and Sørensen [24] noted that dietary interventions can improve growth parameters without necessarily altering the FCR, especially when the energy availability has increased without a corresponding increase in metabolic efficiency [17,24]. The improvement in body-weight gain with the COCO diet suggests that COCO may serve as a valuable dietary supplement to enhance growth performance in broiler chickens. This finding is consistent with previous studies that reported improved energy utilization and growth parameters in poultry receiving diets enriched with MCFAs [5,6].

Metabolomic profiling using HPLC-MS revealed 110,182 features in the plasma, HYP, and DRN, with the HYP exhibiting the highest number of unique features (49,595; 45%). A substantial overlap between the HYP and the DRN (32,832 features; 30%) suggests shared neurochemical activity, which may be indicative of comparable neurotransmitter pathways or more likely due to the fact that both are derived from homogenized brain tissue instead of blood plasma. The limited number of features common to the DRN, HYP, and plasma (6631) underscores a significant metabolic divergence between the central nervous system and peripheral circulation, highlighting the need for direct tissue examination to uncover brain-specific metabolic adaptations [18]. This divergence highlights the importance of direct tissue analysis for understanding changes that occur in the levels or activity of chemicals in the brain, especially those involved in neurotransmission, metabolism, or stress response [25]. The PCA further explained the effects of dietary intervention on metabolomic profiles. The first two principal components (PC1 at 63% and PC2 at 39.3%) provided a strong separation of tissue samples. The plasma samples showed the most pronounced separation between the CON and COCO groups. However, the COCO-treated samples, particularly in the HYP, exhibited a wider spread. This dispersion may reflect adaptive changes in neuroendocrine signaling or stress responses in reaction to dietary modification [21]. The DRN samples displayed even greater variability within the COCO group, which could be due to individual differences in serotonergic regulation or a differential response to dietary lipids [16]. The tissue metabolic profiling revealed the presence of 5193 detected metabolites, with 519 annotated compounds. Among these, 49 metabolites were identified as significant, and five metabolites, succinate semialdehyde, lotaustralin, 2-phenylethyl β-D-glucopyranoside, methyl 4-oxy-2-hydroxy-3,6-dimethylbenzoate, and pleiocarpamine, were found. However, succinate semialdehyde, which was upregulated in the plasma and downregulated in the DRN, is involved in the gamma-aminobutyric acid (GABA) shunt, which is essential for neurotransmitter regulation [9]. The modulation of such metabolites by dietary COCO could potentially influence neurochemical signaling in broilers. Succinate semialdehyde, upregulated in the plasma and downregulated in the DRN, showed that observed differences may reflect tissue-specific modulation of the gamma-aminobutyric acid metabolism associated with neural adaptation, altered energy metabolism via the GABA shunt, or neuroprotective responses to experimental treatments. COCO may improve peripheral energy metabolism and reduce dependence on central GABA shunt activation by providing other sources of energy, such as ketone bodies [9].

Lotaustralin, which was upregulated in the plasma and downregulated in the HYP, may reflect a hormetic response where low levels of a compound trigger the adaptive cellular defense mechanism [16]. In central regions, such as the DRN and HYP, low concentrations of lotaustralin might act as a mild stress signal that upregulates protective pathways, ultimately supporting neuronal health and metabolic balance [16]. 2 Phenylethyl β D glucopyranoside, upregulated in the plasma and downregulated in the HYP, is a glycoside derivative. The presence inside plasma may help in counteracting oxidative stress and systemic inflammation induced by high-energy diets [17]. However, such diets can increase reactive oxygen species and inflammatory cytokines, resulting in systemic stress and metabolic inefficiencies. This glycoside molecule has antioxidant properties that neutralize free radicals and promote the upregulation of the body’s endogenous defense enzymes. These actions foster a more stable internal environment, allowing the broilers to devote greater energy to development and performance instead of addressing physiological stress. Within the HYP, this metabolite might help in stabilizing neurotransmitter systems, thereby influencing appetite regulation and energy homeostasis. Within the DRN, which is an area critical for serotonergic signaling, 2 phenylethyl β D glucopyranoside has the ability to promote neuroprotection by attenuating inflammatory cascades, thus supporting mood and stress responsiveness [18]. The reduction in oxidative damage is essential for preserving neuronal function and may also facilitate better neurotransmitter cycling, which is critical for energy regulation [25]. Methyl 4-oxy-2-hydroxy-3,6-dimethylbenzoate, upregulated in the plasma and downregulated in the HYP, has been shown to function as an antioxidant and neuroprotective agent in poultry. In the bloodstream, Methyl 4-oxy-2-hydroxy-3,6-dimethylbenzoate contributes to reducing oxidative stress and regulating metabolic functions, while in the brain, Methyl 4-oxy-2-hydroxy-3,6-dimethylbenzoate protects neurons [5].

Pleiocarpamine is an indole alkaloid, upregulated in both the plasma and the DRN, with established neuroprotective properties that reduce oxidative stress by neutralizing reactive oxygen species (ROS) that help protect neurons from oxidative damage. This is an indication of potential modulation of the serotonergic pathways in the DRN [5]. The overall effect is an enhancement of neural resilience and improved synaptic plasticity, which together can translate to better performance in broilers [6]. In plasma, the enhanced levels of these compounds may reflect improved systemic antioxidant defenses and metabolic regulation [24]. In the DRN and the HYP, the effects of these metabolites may likely contribute to the maintenance of neurotransmitter balance and protection against neuroinflammation. These reactions are essential for maintaining the physiological and behavioral efficacy of broiler chicks in high-production settings [22,24]. The beneficial properties of these metabolites support the overall advantages of the COCO diet. These compounds may promote growth performance in broilers by enhancing energy metabolism and providing neuroprotective effects [23]. This intricate metabolic regulation suggests that dietary COCO may influence both peripheral and central metabolic pathways as well as the expression of metabolites in the plasma, HYP, and DRN [21,23]. The increased levels of these compounds in the plasma, together with their decreased presence in the DRN and hypothalamus, may indicate a compensatory mechanism wherein improved systemic availability leads to modified central absorption or use [18,26].

Two key pathways were significantly enriched in response to the COCO diet: butanoate metabolism and alanine, aspartate, and glutamate metabolism. Butanoate (or butyrate) metabolism is closely associated with energy production and gut microbial activity. Its enrichment suggests that the intervention might have modulated short-chain fatty acid (SCFAs) metabolism, possibly through microbial–host interactions or changes in energy utilization, particularly in the plasma [20]. The modulation of butanoate metabolism may suggest an interaction between dietary fats and the gut–brain axis, where microbial metabolites influence brain function [8,20,22,23]. The enrichment of alanine, aspartate, and glutamate metabolism enhances the major impact of dietary intervention on amino acid turnover and neurotransmitter cycling. These amino acids play critical roles in the central nervous system (CNS) by participating in the synthesis of neurotransmitters and in maintaining nitrogen balance [17]. The observed shifts in these pathways might be linked to improved neurochemical homeostasis, which, in turn, could affect growth performance and stress responses in broilers [24,25]. The observed changes in amino acid and energy-related metabolic pathways suggest the diet-induced modulation of brain chemistry, most importantly, regions that are involved in metabolic and stress regulation. These may contribute to an improvement in neurochemical homeostasis by stabilizing neurotransmitter levels and reducing oxidative stress. As a result of this, broilers may experience enhanced resilience to environmental stressors, improved appetite regulation, and more efficient nutrient utilization [18]. Conversely, the changes in amino acid metabolism highlight potential neuroprotective and neuromodulatory effects, which are essential for maintaining optimal cognitive and behavioral outcomes in poultry [18].

Amino acids are the main building blocks for the essential neurotransmission needed for the control of cognition and behavior, and their metabolism is crucial to neuroprotection and neuromodulation. Glutamatergic, GABAergic, and serotonergic pathways are significant for keeping the brain stable and working well [27]. In this regard, the metabolism of amino acids produces glutamate, a key excitatory neurotransmitter that promotes learning and synaptic plasticity. It is changed into GABA (gamma-aminobutyric acid) through the GABA shunt, and GABA activity protects neurons from damage and stops neurons from getting too excited [21]. Moreover, serotonin synthesis, which is produced from tryptophan and controls mood, stress tolerance, and hunger in chickens, is directly impacted by amino acid metabolism. Because they may result in better cognitive processing, less sensitivity to stress, and increased feed efficiency, these metabolic adaptations are critical in the production of poultry. The dietary treatments may improve broiler brain function, growth performance, and behavioral flexibility by modifying certain biochemical processes. It is important for chickens to get enough amino acids because they help control the immune system and keep oxidative stress in check in birds’ health in general [21,27]. The distinct metabolomic signatures observed across the plasma, HYP, and DRN emphasize the complexity of metabolic responses to dietary interventions. This discovery opens new avenues for research into how dietary fat types influence brain function and behavior in poultry [16,23].

## 5. Conclusions

Conclusively, COCO supplementation enhanced BWG by improving the growth performance, which might be associated with COCO’s ability to be rapidly absorbed and converted into energy. Additionally, COCO affects the metabolite profile, aiding in neurotransmission and general well-being, while also enriching metabolic pathways critical for growth and brain function. These findings contribute to the growing body of literature on the nutritional and metabolic benefits of MCFAs in poultry production and provide a foundation for future research aimed at optimizing dietary interventions for enhanced performance. Further research is needed to explore the long-term effects of the COCO-supplemented diet to be able to fully understand the multifaceted effects of dietary fats on both systemic and brain-specific metabolic pathways.

## Figures and Tables

**Table 1:** A composition of the experimental diets (%).

Ingredients	CON	COCO
Corn	53.22	53.22
Soybean Meal	39.40	39.40
Fat/Oil	3.00	3.00
Mono-Dicalcium Phosphate	1.81	1.81
Limestone (37%)	0.95	0.95
Salt (NaCl)	0.45	0.45
DL-Methionine	0.35	0.35
NCSU Poultry Mineral Premix	0.20	0.20
Choline Chloride (60%)	0.20	0.20
L-Lysine	0.18	0.18
L-Threonine	0.09	0.09
NCSU Poultry Vitamin Premix	0.05	0.05
Selenium Premix	0.05	0.05
Santoquin	0.05	0.05
**Analyzed Nutrient Composition**		
Metabolizable Energy (Kcal/kg)	1.433	1.432
Crude Protein %	23.06	22.75
Crude Fat %	5.42	5.25
Crude Fiber %	2.1	2.5
Ash %	5.64	5.30
**Calculated Nutrient Composition**		
Total Sulfur Amino Acids %	0.19	0.19
Lysine %	1.44	1.44
Calcium %	0.96	0.96
Available Phosphorus %	0.48	0.48

**Table 2:** The dietary effects of COCO on the growth performance of broiler chickens.

Treatment	CON	COCO	SEM	*p*-Value
BW (Kg/bird)	0.8591	0.8862	0.0729	0.0559
BWG (Kg/bird)	0.8132 ^b^	0.8446 ^a^	0.0083	0.0496
FI (Kg/bird)	0.9661	1.0041	0.0046	0.0698
FCR	1.1882	1.1887	0.0107	0.9610

^a,b^ mean values bearing different superscript letters within a column are significantly different (*p* < 0.05). BW—body weight, BWG—body-weight gain, FI—feed intake, FCR—feed conversion ratio.

**Table 3:** The significant pathways identified and their impact.

Metabolites	Tissue	Profile	Impact on Serotonergic Pathways	References
Succinate semialdehyde	Plasma	Up	Involved in the gamma-aminobutyric acid (GABA) shunt.	[9]
	HYP	*		
	DRN	Down		
Lotaustralin	Plasma	Up	Hormetic response that triggers the adaptive cellular defense mechanism.	[16]
	HYP	Down		
	DRN	*		
2-phenylethyl 3-O-β-D-glucopyranoside	Plasma	Up	Anti-inflammatory and antioxidant properties.	[17,18]
	HYP	Down		
	DRN	*		
	Plasma	Up	Protecting neural tissues involved in stress and behavioral regulation	[5,19]
Methyl 4-oxy-2-hydroxy-3,6-dimethylbenzoate	HYP	Down		
	DRN	*		
	Plasma	Up	Neuroprotective and anti-inflammatory properties and the ROS production pathway	[5]
Pleiocarpamine	Hypo	*		
	DRN	Up		

*—No significant.

**Table 4:** Enrichment pathways and their functions.

Enrichment Pathways	Function in the Brain	References
Butanoate metabolism	Energy production and gut microbial activity. Modulate inflammation and impact immune responses in the brain through inhibiting inflammatory cytokines and regulating immune cell activity. Improve neuroinflammation by suppressing NT-KB activation and controlling microglia and astrocyte cells. Enhances memory and restores cognitive function in mice, suggesting potential benefits in chickens. Preventing the leakage of harmful substances into the bloodstream and affecting the brain. Plays a role in maintaining a healthy gut microbiome, which in turn impacts brain health.	[20,21]
Alanine, aspartate, and glutamate metabolism	Alanine plays a role in nitrogen transport, acting as a carrier of amino groups from the tissues to the liver for urea synthesis. Glutamate and aspartate are the primary excitatory neurotransmitters in a chicken brain, mediating most excitatory synaptic transmission. Crucial for regulating brain functions. Precursor for the synthesis of the inhibitory neurotransmitter GABA	[17,21]

## Data Availability

Original contributions presented in this study are included in the article. Inquiries can be directed to the corresponding author.
